# 2080. Reduced risk of SARS-CoV-2 infection among household contacts with hybrid immunity from recent COVID-19 vaccination and SARS-CoV-2 infection

**DOI:** 10.1093/ofid/ofad500.150

**Published:** 2023-11-27

**Authors:** Melissa A Rolfes, H Keipp Talbot, Kerry Grace Morrissey, Melissa Stockwell, Yvonne A Maldonado, Huong McLean, Karen Lutrick, Natalie M Bowman, Suchitra Rao, Phillip P Salvatore, Hector Izurieta, Jonathan Schmitz, Yuwei Zhu, Kimberly W Hart, Steph Battan-Wraith, Lori S Merrill, Son H McLaren, Ellen Sano, Clea Sarnquist, Sara H Goodman, Joshua Petrie, Karla I Ledezma, Kathleen Pryor, Ayla Bullock, Amy Yang, Jessica E Biddle, Sarah E Smith-Jeffcoat, Sheroi Johnson, Edwin J Asturias, Jessica T Lin, Katherine Ellingson, Edward Belongia, Prasanthi Govindaranjan, Ana Valdez de Romero, Vanessa Olivo, Alexandra Mellis, Carlos G Grijalva

**Affiliations:** Centers for Disease Control and Prevention, Atlanta, Georgia; Vanderbilt University Medical Center, Nashville, Tennessee; Westat, Rockville, Maryland; Columbia University Irving Medical Center, New York City, New York; Stanford University, Stanford, California; Marshfield Clinic Research Institute, Marshfield, WI; University of Arizona College of Medicine, Tucson, Arizona; University of North Carolina, Chapel Hill, North Carolina; University of Colorado School of Medicine, Aurora, Colorado; Centers for Disease Control and Prevention, Atlanta, Georgia; Food and Drug Administration, Bethesda, Maryland; Vanderbilt University Medical Center, Nashville, Tennessee; Vanderbilt University, Nashville, Tennessee; Vanderbilt University Medical Center, Nashville, Tennessee; Westat, Rockville, Maryland; Westat, Rockville, Maryland; Columbia University Irving Medical Center, New York City, New York; Columbia University Irving Medical Center, New York City, New York; School of Medicine, Stanford University, Palo Alto, California; Stanford University School of Medicine, San Jose, California; Marshfield Clinic Research Institute, Marshfield, WI; University of Arizona College of Medicine, Tucson, Arizona; University of Arizona College of Medicine, Tucson, Arizona; University of North Carolina, Chapel Hill, North Carolina; University of North Carolina, Chapel Hill, North Carolina; Centers for Disease Control and Prevention, Atlanta, Georgia; Centers for Disease Control and Prevention, Atlanta, Georgia; Centers for Disease Control and Prevention, Atlanta, Georgia; CU School of Medicine, Aurora, Colorado; University of North Carolina at Chapel Hill, Chapel Hill, North Carolina; University of Arizona, Tucson, Arizona; Marshfield Clinic Research Institute, Marshfield, WI; Stanford University School of Medicine, San Jose, California; Columbia University Irving Medical Center, New York City, New York; Westat, Rockville, Maryland; Centers for Disease Control and Prevention, Atlanta, Georgia; Vanderbilt University Medical Center, Nashville, Tennessee

## Abstract

**Background:**

COVID-19 vaccines reduce the risk of symptomatic SARS-CoV-2 infection, but it is unclear the extent to which vaccines or prior infection reduce the risk of infection in high transmission settings like households.

**Methods:**

We screened individuals who tested positive for SARS-CoV-2 (index cases) recruited at 7 sentinel testing sites and through a nationwide effort during Sep 2021–Apr 2023. Index cases and their households (HH) were enrolled ≤6 days after the index case’s illness onset. Household contacts (HHC) had daily self-collected nasal swabs or saliva samples tested by RT-PCR for SARS-CoV-2. We determined COVID-19 vaccination status by plausible self-report (with date) or vaccination records, and prior SARS-CoV-2 infection by self-reported prior positive test (with year) or anti-nucleocapsid antibodies assessed at enrollment. We considered HHC with prior COVID-19 and ≥2 COVID-19 vaccine doses as having “hybrid immunity”, and assessed the effects of ≥2 vaccine doses, prior COVID-19, or hybrid immunity on the risk of PCR-confirmed SARS-CoV-2 infection among HHC by GEE Poisson regression adjusted for age of the HHC, recruitment strategy, household density (ppl/bedroom), and enrollment month.

**Results:**

We included 1,324 HHC (Fig 1); 73% enrolled May–Nov 2022, when Omicron BA.4/5 predominated; 28% were aged < 18 years (Table 1). Most (89%) had some immunity to COVID-19: 54% from vaccination only, 7% from infection only, and 26% from hybrid immunity. Most HHC without immunity to SARS-CoV-2 were children (64%). Of HHCs, 61% became SARS-CoV-2 positive during follow-up. In a model that accounted for all sources of immunity (figure 2), prior vaccination or prior infection alone did not provide significant protection, only HHC with hybrid immunity had significantly reduced risk of infection (adjusted relative risk: 0.80, 95% confidence interval: 0.68, 0.94; Fig 2). The risk of infection was lowest (43%) when the HHC’s last immunizing event (vaccination or infection) occurred ≤6 months before the index case’s illness onset.

Eligibility and inclusion of HH contacts in analysis of COVID-19 transmission

**Figure:**
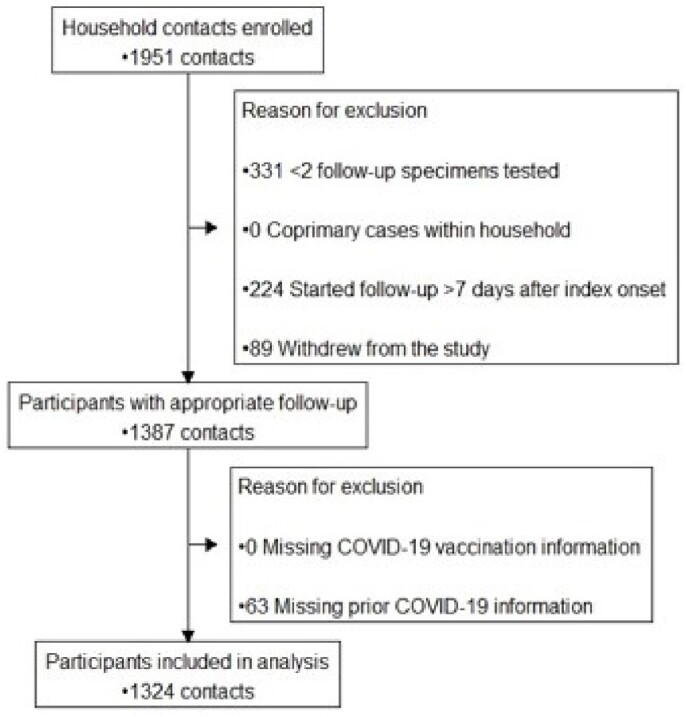

Figure 1

Table 1

**Figure:**
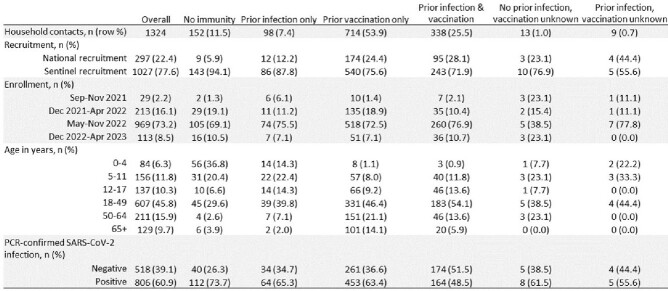

Demographic characteristics of HH contacts by COVID-19 vaccination or prior COVID-19

Figure 2

**Figure:**
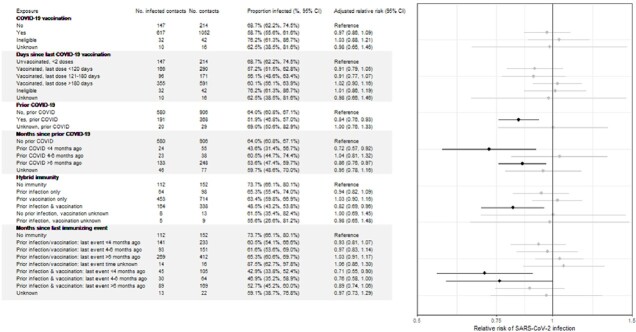

Adjusted relative risk of infection among HH contacts by COVID-19 vaccination or prior COVID-19

**Conclusion:**

The risk of SARS-CoV-2 infection among HHCs continues to be high. While vaccination alone was not effective at preventing SARS-CoV-2 infection, HHCs with hybrid immunity from recent vaccination or prior infection had the lowest risk of infection.

**Disclosures:**

**Yvonne A. Maldonado, MD**, Pfizer: Grant/Research Support|Pfizer: Site Investigator, DSMB member **Huong McLean, PhD, MPH**, Seqirus: Grant/Research Support **Suchitra Rao, MBBS, MSCS**, Sequiris: Advisor/Consultant **Joshua Petrie, PhD**, CSL Seqirus: Grant/Research Support **Edwin J. Asturias, MD**, Hillevax: Advisor/Consultant|Moderna: Advisor/Consultant|Pfizer: Grant/Research Support **Edward Belongia, MD**, Seqirus: Grant/Research Support **Carlos G. Grijalva, MD, MPH**, AHRQ: Grant/Research Support|CDC: Grant/Research Support|FDA: Grant/Research Support|Merck: Advisor/Consultant|NIH: Grant/Research Support|Syneos Health: Grant/Research Support

